# The Application of Extraperitoneal Ostomy Combined with Pelvic Peritoneal Reconstruction in Laparoscopic Abdominoperineal Resection for Rectal Cancer

**DOI:** 10.1155/2019/3015958

**Published:** 2019-02-05

**Authors:** Sen Wang, Qingyang Meng, Jun Gao, Yuqin Huang, Jie Wang, Yang Chong, Youquan Shi, Huaicheng Zhou, Wei Wang, Dong Tang, Daorong Wang

**Affiliations:** ^1^The First Clinical Medical College of Nanjing Medical University, Nanjing, Jiangsu 210029, China; ^2^Department of General Surgery, General Surgery Institute of Yangzhou, Northern Jiangsu Province Hospital, Clinical Medical College, Yangzhou University, Yangzhou, Jiangsu 225000, China; ^3^Dalian Medical University, Dalian, Liaoning 116044, China

## Abstract

**Background:**

Due to the technical difficulty, it is not common to close the pelvic peritoneum in laparoscopic abdominoperineal resection (LAPR) in China, which increases the risk of related complications. Permanent sigmoid colostomy is performed through the transperitoneal route conventionally in LAPR. This leads to the high occurrence of parastomal hernias and bowel obstructions. To prevent the complications and reduce surgical costs of LAPR, we performed some modifications for it.

**Methods:**

38 patients diagnosed with low rectal cancer during July 2014 to July 2016 received LAPR with our modifications. First, the mobilization of the rectum and lymphadenectomy were identical to the classical routine method. Second, two sutures were performed on the pelvic peritoneum with the first to reduce the tension, followed by the second continuous suture to close the pelvic floor. Third, a tunnel was made between the parietal peritoneum and abdominal wall for the end sigmoid to pass through to finish the colostomy.

**Results:**

LAPR was performed on totally 38 patients successfully with no case transferring to open surgery. The follow-up period was from 1 month to 1 year. The mean operative time was 142.2 ± 16.5 min ranging from 100 min to 175 min. The mean hospital stay was 12.0 ± 1.5 days. No case underwent the reconstruction of stoma. There was not a single complication of LAPR with these two techniques that occurred to all 38 patients.

**Conclusion:**

We consider LAPR with our two techniques feasible and safe, which can be accepted quickly to improve the life quality of patients. Therefore, we suggest our procedures as the first choice during LAPR surgery. This trial is registered with trial registration number 2014028.

## 1. Introduction

Since the incidence of colorectal cancer has ranked no. 3 among all malignant tumor diseases in the globe, abdominoperineal resection (APR) with the colostomy is now still one of the radical treatments in 24-38% patients with low rectal cancer [1]. As we know, laparoscopy technology has gained its great popularity in surgery and is now widely accepted in the colorectal cancer treatment. It is proved to be as effective and efficient as open surgery in terms of safety, time, and oncological clearance, making laparoscopic surgery the first option instead of open in many cases [2]. When it comes to laparoscopic abdominoperineal resection (LAPR), however, although the surgery has already been quite established, there are some techniques that need evolving. Due to the very difficult technique, it is not common to close the pelvic peritoneum in laparoscopic surgery in China while it is a standard procedure in open surgery [2]. This may cause related complications which affect the life quality of patients. The alternative option for laparoscopic pelvic floor reconstruction is the biological mesh, but there are only a few reports on it [3–7]. Meanwhile, in most LAPR, permanent sigmoid colostomy is performed through the transperitoneal route, in which way it leads to the high occurrence of parastomal hernias and bowel obstructions. In some cases, parastomal hernia was considered to be inevitable [3–7]. Due to these disadvantages, modifications are required for the current surgical procedures. Therefore, laparoscopic extraperitoneal colostomy and laparoscopic closure of the pelvic peritoneum have become the solutions to these problems, even though they are applied by very few surgeons because of the demanding surgical skills [1]. We modified these two techniques in LAPR with our own methods and achieved satisfied outcomes in our center.

## 2. Patients

From July 2014 to July 2016, we performed LAPR with our modified techniques on 38 patients with low rectal cancer at the Northern Jiangsu Province Hospital. The 38 cases included 26 male and 12 female patients with a mean age of 60.1 ± 12.0 ranging from 37 to 89 years. The diagnosis for all the patients was low rectal carcinoma less than 5 cm from the anal verge with pathological sections through the colonoscopy and computed tomography. Among all the 38 patients, the pathological types included 20 tubular adenocarcinoma, 8 papillary adenocarcinoma, 8 mucinous adenocarcinoma, 1 epidermoid carcinoma, and 1 signet ring cell carcinoma. In terms of T staging, 10 were classified into the T2 group while 17 in the T3 group and 11 in the T4 group. Low-fiber diets and intestinal lavage powder were orally administrated three days and one day before surgery, respectively. Antibiotic prophylaxis was administrated 30 minutes before the induction of anesthesia. Radiation therapy and neoadjuvant chemotherapy were administrated in ten T4 patients and seventeen T3 patients before operations.

## 3. Methods

Patients underwent endotracheal intubation under general anesthesia, placed in the dorsal lithotomy position, and followed by the establishment of pneumoperitoneum with the range from 12 to 15 mm Hg (1 mm Hg = 0.133 kPa). Surgical procedures of laparoscopic mobilization of rectal tumors were identical to a classical routine following the total mesorectal excision (TME). We adopted the five-port approach. The first 10 mm trocar was inserted 2-3 cm above the umbilicus for the camera. A 12 mm trocar and 5 mm trocar were inserted 4-5 cm proximal from the right anterior superior ilium spine and at the joint position of the level of umbilicus and right midclavicular line, respectively, for the main operator. On the left side, two 5 mm trocars were inserted at the reverse Mcburney point and 3-4 cm away from the umbilicus at the umbilicus level, respectively, for the assistance. Inspections should be made first on the organs in abdominal and pelvic cavities. Then the peritoneum of the sigmoid and rectum was opened by a harmonic scalpel to move into the Toldt space. We extended the space by dissecting towards the vertical and left directions along the Toldt fascia with paying attention to protecting both ureters. Vessels of mesentery were resected after the lymph codes and fat tissues around were dissected. The outside peritoneum of sigmoid and rectus were opened through with the extension from the descending colon to the pelvic floor. Presacral space was cut open, and deep pelvic cavity was then reached with attention to protect the iliohypogastric nerve. The peritoneal fold and ligaments were then cut open followed by the dissection of the rectum.

**Figure 1:**
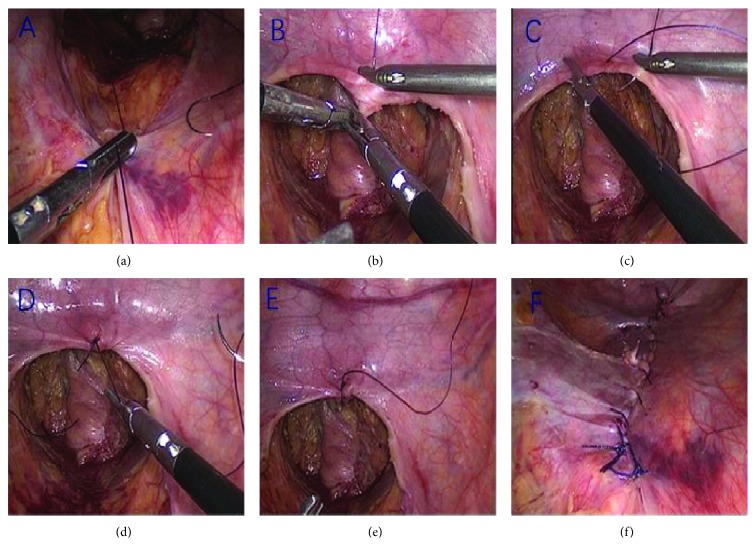
(a) The first square knot which needed force and care. (b–e) The second figure-of-eight suture. (f) The continuous suture was then performed to close the gap.

The difference started from the blunt dissection of the pelvic rectum. Our method is about to combine the laparoscopic closure of the pelvic floor and extraperitoneal ostomy as a consistent procedure. Mobilization of the pelvic peritoneum around the rectum should be as little as possible on the basis of oncological clearance; otherwise, it would be more difficult to close the pelvic floor for the lack of peritoneum. After gentle mobilization of the rectum, ATS endo GIA was used to cut it. The specimen was then pushed into the minor pelvis until it was taken out along with the pelvic exenteration. Following that, the perineal team and abdominal team began to perform the separate surgeries synchronously. The perineal team did the classical routine method of pelvic exenteration accordingly while the abdominal team performed the laparoscopic closure of the pelvic floor. The first suture was at the peritoneal folder. The needle forceps were used to suture from the right side to the left side, followed by the first square knot which needed force and care to keep the balance of making it tight and intact with the assistant's help to clamp the knot (Figure 1(a)). The two more knots were followed to make sure it was tight. The second figure-of-eight suture of pelvic peritoneal was on the furcation of the iliac artery with Vicryl 3-0 which closed the peritoneum at the entrance to the upper pelvis (Figure 1(b)). The first stitch was at the top position of the entrance, followed by the right and left stitches which both were 1.5 cm away from the original position, forming the figure eight to close the top middle 3 cm peritoneum (Figure 1(c)). It was not easy to avoid the defect, so it should be close to the retroperitoneal tissue, leaving no pores, in order to prevent postoperative internal hernia caused by the small intestine. The figure-eight suture made the perineum besides the second stitch stay close and covers each other vertically (Figure 1(d)) which required comparatively bigger force. This suture reduced the tension of pelvic peritoneum greatly. The Vicryl material was left 5-6 cm in one end for backup after knotting (Figure 1(e)). The continuous suture was then performed to close the gap. The assistant's tight hold continually plays an important role to keep the peritoneum fully closed while the main operator was suturing (Figure 1(f)). When the operator was suturing, the assistant needed to hold the material tight to keep the previous continuous suture tight. When the operator was straining the suture material, the assistant gradually loosened it. This operator-assistant switch was performed every two stitches. After the closing procedure, a careful inspection was performed to see if there were any small gaps in the peritoneum or any bowel tissues which were stuck in them. Since both teams were working synchronously, the procedure of the abdominal team should be kept ahead of that of the perineal team under the surgeon's control, which in this way, pneumoperitoneum would not be needed to reestablish because the ahead-of-schedule closure of the pelvic peritoneum would maintain the state of pneumoperitoneum if there was no obvious air leakage from the closure. This could be one criteria of the quality of closure because if there were any obvious leakages between the sutures, the pneumoperitoneum would collapse that we would notice. If there were only small leakages that have no obvious effects on the pneumoperitoneum, continuing the surgery without reestablishment of the pneumoperitoneum would not be a problem.

**Figure 2:**
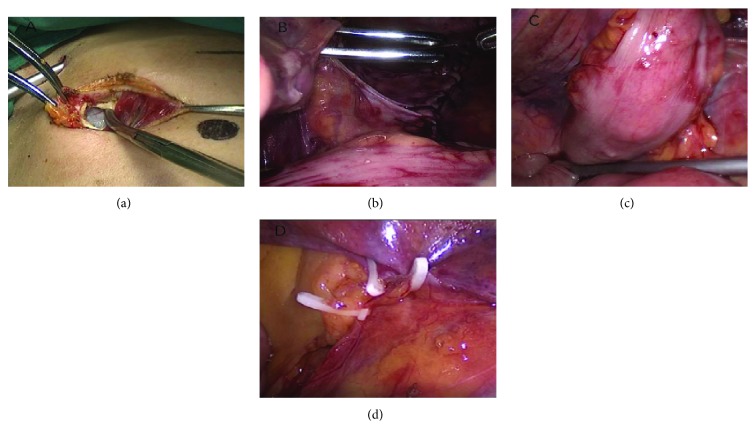
(a) A preplanned round skin incision of 3 cm in diameter should be made at the left McBurney point. (b) Break through the peritoneum to clamp the end sigmoid. (c) The end sigmoid was then grasped and exteriorized slowly through the oblique tunnel. (d) Close the space between the visceral peritoneum and stump sigmoid.

Laparoscopic extraperitoneal colostomy was then performed by the abdominal team. A preplanned round skin incision of 3 cm in diameter should be made at the left McBurney point, below which is the outer leaf of the rectus abdominis. The skin and subcutaneous tissues of this incision were resected. Through this stoma, blunt dissections by Kocher forceps were made to break open the aponeurosis of the obliquus externus abdominis with a cross incision on it. The musculus obliquus internus abdominis was then cut by the forcep holder to expose the peritoneum without incising it (Figure 2(a)). The main operator's left index and middle fingers inserted the stoma into the space between the musculi obliquus internus abdominis and peritoneum to tunnel down the joint position of the descending colon and sigmoid and expand the tunnel to 2-3 fingers width. The sigmoid colon should be pulled 3 cm out of the peritoneal cavity by this tunnel, to ensure good blood supply, tension-free, no torsion, and not oppressed [8]. The impression of extraperitoneal fingers would remind us whether the tunneling site reach the joint position. Intra-abdominal right forceps would help move the visceral peritoneum to get the fingers into position because two fingers would not insert deep enough. When the position was confirmed by the fingers, the assistant's extraperitoneal sponge forceps were inserted along with the two fingers to break through the peritoneum to clamp the end sigmoid (Figure 2(b)). It was then grasped and exteriorized slowly through the oblique tunnel with paying attention not to twist the mesentery which will cause bowel obstruction. During the procedure, the joint bowel of the descending colon and sigmoid must be placed onto the visceral peritoneum leaving no space between the bowel and the visceral peritoneum that surrounded the incision (Figure 2(c)). Finally, clasps could be used to close the space between the visceral peritoneum and stump sigmoid, which would prevent bowel incarceration here (Figure 2(d)). The pneumoperitoneum was still almost totally maintained after the forcep's breaking through the peritoneum, which would not affect our surgical process [9]. After the end sigmoid was drag out through the tunnel, two and a half layers of sutures were needed to fix the end sigmoid which resembled the regular colostomy. The first half layer was the continuous suture of the end sigmoid and parietal peritoneum which is located at the end sigmoid 1 cm from the skin surface. Since the end sigmoid is between the parietal peritoneum and abdominal wall, the suture can only be performed on the half perimeter of the sigmoid representing the half layer. The second layer consisted of the surrounding sheath of the musculus obliquus externus abdominis through the stoma suturing with the end sigmoid which is about 0.8 cm from the skin surface. This continuous suture was performed along the perimeter making it a full layer. The third layer consists of the continuous transfixed suture between the dermal layer and the sigmoid to finish the colostomy. These two and a half layers would prevent the stoma from retraction. Lastly, we still need two fingers with paraffin oil to inspect the tunnel including the peritoneal turning angle from through the colostomy stoma to see if there were any narrows in the tunnel.

## 4. Results

LAPR was performed on 38 patients successfully with no case transferring to open surgery. Laparoscopic closure of the pelvic peritoneum and extraperitoneal colostomy were performed in all 38 patients. The mean operative time was 142.2 ± 16.5 min ranging from 100 min to 175 min. The mean hospital stay was 12.0 ± 1.5 days. The mean intraoperative bleeding was 74.7 ± 50.1 ml ranging from 20 ml to 200 ml. No intraoperative blood transfusion was made during operations. The follow-up period was from 1 month to 1 year. According to Akamoto et al. [9], complications within 4 weeks were defined as short-term complications such as hemorrhage, bowel ischemia, and edema. After 4 weeks, they were defined was long-term complications such as parastomal hernia, stomal retraction, bowel narrow, and obstructions. One case of recurrence of carcinoma was located on the rectum one year later and dealt with reoperation. No case underwent the reconstruction of the stoma. There was not any single complication of LAPR with these two techniques, including both short- and long-term complications of bowel adhesion, infections, ischemia, necrosis, parastomal hernia, perineal hernia, and retraction that occurred to any of these patients. No radiation therapy was administrated after surgery for all cases. This incredible achievement indicates that our modified procedure has showed its great advantages on the patients.

## 5. Discussion

With the rapid developments and popularity of laparoscopic technology in the colorectal cancer, APR surgery has gained much more attention than ever before. Some surgical procedures that are used to be considered not feasible in laparoscopic surgery may need reconsideration today.

In the open APR surgery, the pelvic peritoneum is supposed to be closed, as a standardized procedure to prevent postoperative complications, such as perineal hernia and radiation-induced enteritis [2]. Even though perineal hernia is a very rare long-term complication as the incidences are less than 1% after conventional APR and unknown after laparoscopic APR, respectively [3], preventive measures still have to be taken. Biological mesh has been used to reconstruct the pelvic floor to prevent the intra-abdominal contents like small bowel from protruding through the defects in the pelvic floor [3], which will protect bowel from the adhesion, obstructions, and the radiation defects if postoperative radiation therapy was performed in the low position. Capillary obliteration and fibrosis would be caused by radiation therapy, leading to altered immune mechanisms [10]. However, complications after biological mesh such as infections, fistula, and erosion are also problems, not to mention the extra high costs [3, 5, 10, 11]. On the other side, due to the technical difficulty, most surgeons may choose not to suture the pelvic peritoneum in laparoscopic surgery [3]. As we know, knotting and suturing in laparoscopic surgery take a lot more time and efforts than in the open surgery. While in a LAPR, a great tension of a separate pelvic peritoneum makes it even harder to close it with a certain degree of dragging force in case of damaging the pelvic peritoneum. The techniques of laparoscopic closure of the pelvic peritoneum turn out to be the key to this problem.

During our method, the previous two sutures were the critical parts of closing pelvic peritoneum. Performing in these two positions eased the difficulty of suturing compared with a single continuous suture directly all the way without the previous two because it decreased the tension of the pelvic peritoneum between the first suture and the second suture so that the following continuous suture would become much easier. In this way, we did not need other particular surgical instruments but only common needles and forceps. The preparation for the suture was that more peritoneum needed to be saved during mobilization in case of lacking it, whereas if the tension was really too high to hamper the closure, surgeons should evaluate the feasibility and make the proper decision whether to quit the closure. Until now, no case in our center has been transferred to nonclosure surgery for the difficulty reason. Since the abdominal and perineal teams were both working synchronously, this closure procedure would not cost extra time because it was included in the time of pelvic exenteration. Our method saved not only the time of laparoscopic closure of pelvic peritoneum but also the time of pneumopeirtoneum reestablishment, so the total surgical time did not increase significantly. Although we did not have another comparison group of nonclosure of the pelvic peritoneum with transperitoneal colostomy surgery for evidence support, our mean operative time was still satisfying.

The conventional way of a transperitoneal route has comparatively high incidents of long-term complications, such as parastomal hernias [12], stomal prolapses, and stomal retraction. It is reported that half of the patients who received transperitoneal colostomy may have some degree of parastomal hernia [12]. Due to the tension of retracting, the stoma on the abdominal walls may be extended, creating more space between the stoma and the stump sigmoid end, which can also contain small bowel tissues through the incision on the peritoneum. Prophylactic stoma mesh is now also widely accepted as the solution to parastomal hernia repair by western surgeons. The main purpose of this technique is to use the stoma mesh to overlay the fascial defects to decrease the incidents of parastomal hernia. However, the latest research revealed that prophylactic stoma mesh could not prevent parastomal hernias as we expected [13]. This study showed that patients of colorectal cancer with and without prophylactic mesh after APR did not demonstrate a significant difference in the incidence of parastomal hernias. Furthermore, infections and erosions of small bowel were associated with the stoma mesh. Thus, it is possible for patients to save the expenses of stoma mesh because the necessity of applying it may become debatable. Secondly, since the stump was made vertical to the peritoneum and abdominal walls, the space between the stump and peritoneum may accommodate tissues of bowel, forming obstructions. On the contrary, the construction of extraperitoneal colostomy kept the stump between the parietal peritoneum and abdominal walls parallel in the tunnel. The sigmoid's passing through the tunnel avoids the disturbance of intra-abdominal bowel at utmost. The peritoneum provides with reinforcement to keep the stump stay stable onto the walls and prevent it from retracting to pelvic cavity, therefore leading to low incidents of stoma prolapses. Along with lower retraction force, the incidence of parastomal hernia will also decline because the stoma is least likely to be expended. Thus, the extraperitoneal route decreases the incidents of these long-term complications which corresponds to the previous reports [14, 15]. Through the extraperitoneal route, the sigmoid is supposed to form a gradual curvature at the incision of the visceral peritoneum, which assembles the original physiological curvature. However, there is a possibility for the stump sigmoid to shape a sharp angle, which will also lead to bowel obstruction. Therefore, this requires our special attention to place the bowel properly in the junction of the peritoneum of the lateral wall and pelvic floor to leave no space between them with no twists of bowel. To prevent the situation that the tunnel was stressed too much by the peritoneum, fingers needed to be inserted to check the diameter of the tunnel to make sure that bowel can pass smoothly. From the angle of anatomy, nerves of parietal peritoneum which come from intercostal nerves and subcostal nerves of parietal peritoneum are quite sensitive to the pain caused by the mechanical and chemical stimulation. Since the end sigmoid stays onto the parietal peritoneum, the stimulation of human excreta would be sensed by the peritoneum nerves. The pain before defecation would help patients form a new defecation reflection gradually, which would improve the life quality of patients.

Although our method showed great advantages, there are still some limits that we may not ignore. Firstly, the size of our sample was too small that may hide the incidents of complications. More LAPR surgeries need be performed to enrich the sample size. Moreover, the data of traditional transperitoneal colostomy with a nonclosure pelvic peritoneum also needs to be collected for the comparison for more evidence. Longer follow-up shall be also conducted to investigate the incidence of complications as a one-year follow-up is comparatively short. As far as the results are concerned, although we achieved good outcomes of complications, the mean hospital stay is still longer than we expected, which probably means our technique is more invasive to patients. More advancements shall be made to ameliorate our technique.

Despite of these drawbacks, our surgical results demonstrate satisfying outcomes. Laparoscopic closure of the pelvic peritoneum and extraperitoneal colostomy greatly decrease the incidents of short- and long-term complications. Most of all, these techniques are simple, safe, and easy to learn, which can be accepted quickly to improve the life quality of patients. Therefore, we suggest our procedures as the standardized procedure during LAPR surgery.

## Data Availability

The [DATA TYPE] data used to support the findings of this study are included within the article.

## Supplementary Materials

Supplementary Materials The clinic characteristics of 38 patients with low rectal cancer (original data).
